# Epiphytic Bryophytes in an Urban Landscape: Which Factors Determine Their Distribution, Species Richness, and Diversity? A Case Study in Wroclaw, Poland

**DOI:** 10.3390/ijerph19106274

**Published:** 2022-05-21

**Authors:** Ludwik Żołnierz, Ewa Fudali, Mariusz Szymanowski

**Affiliations:** 1 Department of Botany and Plant Ecology, Wrocław University of Environmental and Life Sciences, pl. Grunwaldzki 24a, 50-363 Wrocław, Poland; ewa.fudali@upwr.edu.pl; 2 Institute of Geography and Regional Development, Faculty of Earth Sciences and Environmental Management, University of Wroclaw, pl. Uniwersytecki 1, 50-137 Wrocław, Poland; mariusz.szymanowski@uwr.edu.pl

**Keywords:** urban bryoflora, urban heat island, urban environment

## Abstract

There is still a lack of knowledge on the effect of urban environmental factors on bryophyte species distribution and richness. The goal of this study was to fill that gap. We assumed the hypothesis that the urban heat island is the most important factor affecting epiphytic bryophyte species in urban space. The survey was based on a network of 500 one hectare study plots, scattered throughout the city of Wrocław (SW Poland). A set of 27 environmental factors was assessed in the field, as well as by the collection, processing, and interpretation of satellite imagery, LiDAR scans, and climatological data. Canonical correspondence analysis was used to evaluate the significance of the effect of the studied variables on the distribution of bryophyte epiphytes. The effect of the normalized difference vegetation index on epiphytic bryophyte distribution and richness was the strongest. The effects of the urban heat island as well as the tree species diversity appeared weaker, though significant. Among the tree stands features, the supply of European ash *Fraxinus excelsior* and tree height appeared to be the strongest. Maintaining afforested areas rich in old tree individuals with cooler and more humid microclimates seems to be crucial to the keeping of epiphytic bryophyte species diversity in the urban landscape.

## 1. Introduction

Bryophytes can easily be overlooked as an element of urban greenery and biodiversity since they produce relatively little biomass and occupy specific niches. In spite of this, their presence in the urban area can also be seen as beneficial for human health. They may be considered as a potential natural air filter in relation to harmful trace elements and urban airborne polycyclic aromatic hydrocarbons, which has recently been documented [1]. Bryophytes growing on tree trunks seem to be particularly predisposed to this role because they are in full contact with air and absorb all substances from it [2]. Moreover, bryophytes are photosynthetically active all year round, thus this green filter can also work in winter when other elements of urban greenery die off or fall into hibernation. Currently, in many European urban areas there is a visible trend of installing green walls or other constructions containing moss cultures designed to filter the air in city centers (see the “City Tree project”). There is also another aspect of the bryophytes’ potential influence on human health. They are an important component of the biological diversity in the urban ecosystem [3,4], increasing the sense of aesthetics, reducing stress, and thus improving the psychological comfort of residents. Bryophytes can bring aesthetical values important for mental balance. Various studies have indicated that the bryophyte flora of various cities, regardless of their size, may include more than 100 species ([5] and the literature quoted therein). Moreover, cities often contain bryophyte species that are regionally rare or endangered.

Bryofloristical data gathered over the last two decades of the 20th century noted that epiphytic bryophytes in urban areas only occurred in large parks and urban forests [6]. Presumably, therefore, epiphytic bryophytes were not taken into account in the context of research on the influence of plants on the air condition in cities, although the ability of mosses to accumulate heavy metals from the dust deposition was already well-known and studied [7]. However, at the beginning of the 21st century, the return of epiphytic mosses to city centers was recorded in some European cities, such as London [8], Lisbon [9], and Katowice [10]. The cited research reported an increase in epiphytic species richness and the number of their localities. Although interest in studies on the urban epiphytic bryoflora is increasing, still little is known about what factors determine their current distribution in cities. Only a few studies have examined the effects of anthropogenic environmental factors such as the variability of land management methods [6,11,12] or distance from sources of air pollution [8,9] on the distribution of urban epiphytic bryophytes. To date, urban heat islands (UHIs) have not yet been studied as potential drivers of epiphytic bryophyte occurrence in cities. Although Oishi [3,4] recently highlighted the significant effect of the urban heat island phenomenon combined with drought stress on bryophyte species richness in historical Japanese moss gardens, these studies concerned mosses growing on the ground or stones, not epiphytes.

The specificity of epiphyte biology relates to the diversified and changeable ability of many bryophyte species to colonize the trunks of certain tree species. This implies that only some of the latter are inhabited by a large number of bryophyte species, while the trunks of many other tree species are poor in this regard. Such relationships are not constant throughout the entire geographical ranges of individual bryophyte species, and it was observed that a change in the preferred host tree species is dependent on climatic conditions and the availability of phorophytes [2,13]. Although host tree species richness and supply in cities have rarely been considered and quantitatively analyzed as factors determining epiphytic bryophyte distribution [12], some data and general conclusions suggest that the richness of epiphytes in a city depends on the variety of phorophytes [10,11]. These opinions coincide with the results of research and analyses conducted on epiphytes in Central European forests, which showed the positive effect of tree species diversity on the number of epiphytic bryophyte species [14,15,16,17].

These issues represent an important gap in our knowledge that should be filled to understand the relationships between epiphytic bryophyte species and the urban environment. As such, addressing these issues is of crucial importance for urban biodiversity conservation.

Various authors have emphasized the significance of urban green space heterogeneity [18,19] and the need for differentiated management as a way to maintain habitat variability to ensure the preservation of high biodiversity [20]. Such studies may also be useful in terms of creating a healthier environment in cities. Knowing what factors affect the distribution of epiphytic bryophytes would make it possible to shape the urban environment in such a way that it would favor the settlement of epiphytes and thus increase the surface of this natural absorber of dust and harmful substances. As a result of cleaner air, the health of inhabitants would also improve.

We tested the hypothesis that the distribution and richness of epiphytic bryophyte species within urban spaces are strongly related to the UHI phenomenon. We assumed that UHIs are conditioned by the structural diversity of the urban landscape and the various patterns of land use. We also assumed that the occurrence of some epiphyte species may be more strongly related to the presence of a specific host tree species than to the influence of climatic factors.

The present study addressed the following questions: (1) Which of the environmental factors studied here determine the species distribution, richness, and diversity of epiphytic bryophytes within a large city? (2) Assuming that the epiphytic bryophyte species are often closely related to the particular tree species we aimed to reveal, do the species number and diversity of trees affect the species number and diversity of epiphytic bryophytes in a city?

## 2. Materials and Methods

### 2.1. Study Area

**Figure 1:**
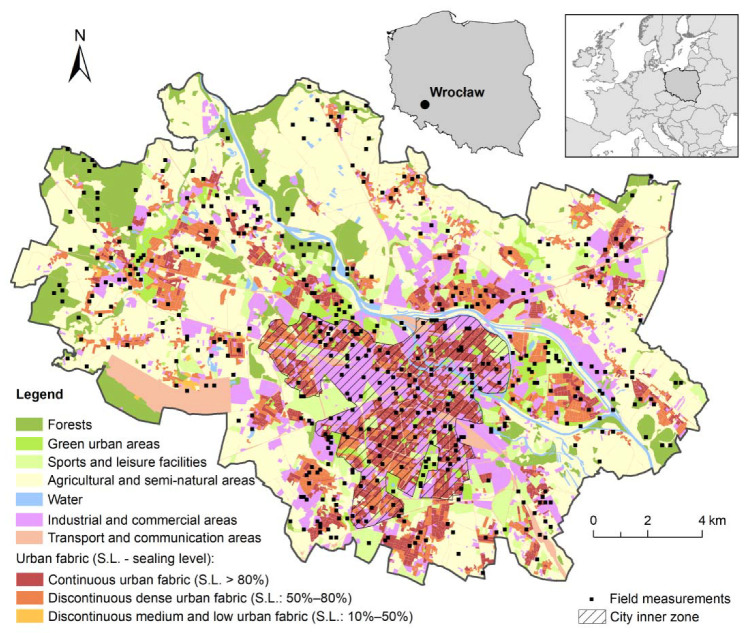
Urban landscape elements, land use, and the distribution of study plots in the city of Wroclaw area. The reader is referred to the online version of this article for interpretation of the contents expressed in color in this and further figures.

The study was carried out within the administrative area of the city of Wrocław (Figure 1). This is the fourth-largest city in Poland in terms of population (about 640,000), encompassing an area of 292 km^2^. Wrocław lies on the Odra river in southwestern Poland (51°6′ N; 17°2′ E) within an altitude range of about 100–140 m a.s.l.

The city is located in a midlatitude, temperate, transitional (maritime–continental) climate zone characterized by a high frequency of polar air masses and a dominating western flow. The mean annual temperature is about 9 °C and the average sum of precipitation is slightly less than 600 mm. The rainfall regime is dominated by continental features, with maxima occurring in July [21]. The climate of Wrocław is also a typical urban climate [22,23] with a clearly marked UHI, lower relative air humidity, less solar radiation, and a lower average wind speed. Notably, these features are more pronounced in the central areas of the city.

The average annual magnitude of the UHI in the densely-built up center of Wrocław is about 1.0 °C, but, as expected, is lower in large housing estates of tall concrete buildings (0.7 °C) and residential areas of low estate houses (0.3 °C). The maximal intensity of the UHI in Wrocław can exceed 10 °C; however, large intensity UHIs (>5 °C) are evident only during <4% of night hours and on very few occasions during the daytime. More detailed characteristics of Wrocław UHIs are discussed in [24].

Wroclaw is one of the few Polish cities whose urban climatic environment has been widely studied and described in recent years. Studies by [24,25] revealed that the processes shaping the city’s climate are consistent with those described in other major urban areas around the world. This prompted us to study the impact of specific urban climate factors on the distribution and richness of the epiphytic bryophyte species.

Wrocław was founded in the 10th century, and it therefore has a spatial structure similar to other European cities established in the medieval period. A compactly built-up downtown encompasses about 30% of the city area. Overall, the city comprises the old downtown, factories, large housing estates built mostly during the 1960s–1990s, and a strongly developed network of streets. About 17% of the downtown is occupied by urban greenery, consisting of four large parks and a few smaller, wooded sports facilities, cemeteries, and walking routes. Almost 45% of the city area has an agricultural character, with scattered buildings between gardening farms, meadows, and cultivated fields (Figure 1). In the last few decades, the latter have been partly fallowed and recently systematically become built-up into residential estates.

### 2.2. Data Sampling

A total of 500 study plots (100 × 100 m square) were established within the administrative area of the city (Figure 1). Initially, we divided the entire city area into a 100 × 100 m grid. To ensure that the research was carried out in plots with varying degrees of tree cover, we used a stratified random sampling scheme. We preceded this by calculating the tree cover in each grid, for which we used the LiDAR-based digital surface model. Then, we divided all of the grids into deciles depending on the tree cover and randomly selected 50 plots in each decile. Sampling was performed using the random selection within subsets tool included in Hawths Tools (spatialecology.com, accessed on 15 April 2013), which is a free extension of ArcGIS.

### 2.3. Field Surveys

Field surveys were carried out during the years 2013–2016. Study plots were located in the field using orthophotomaps and a GPS receiver. Where necessary, the corners of the plot were marked with geodetic poles. In each plot, the number of tree species and the number of individual trees of each species with a diameter above 10 cm were counted to estimate the potential host tree supply. The trunk of each studied tree was examined for the presence of bryophytes at 0.8 m above ground level and above. This limitation was due to the fact that the lower parts of tree trunks are often overgrown with competing ground bryophytes [2,12]. If bryophytes did occur, all species occurring in the 0.8–1.2 m sector were recorded according to the procedure given by [12]. Most of the bryophyte species were identified in the field. Doubtful or unknown species were collected and then identified via the use of a microscope in the laboratory. Since the bryophyte species living on tree trunks also have varying colonization abilities in other substrates (e.g., soil, rotten wood, and rocks), two types of epiphytes are distinguished in the context of bryophyte ecology: obligatory (inhabiting only tree trunks) and facultative (inhabiting trees and other substrates) [2]. The latter includes epiphytic–epilithic species that colonize tree trunks and rocklike habitats as well as multisubstrate epiphytes that can grow on three or more substrates [26]. Classifying a species as an obligatory or facultative epiphyte always relates to regional conditions [7]. In the present study, the species affiliations (obligatory or facultative epiphytes, see Appendix A) were based on bryological data published for Wrocław and its vicinity to date ([26], and literature quoted therein).

### 2.4. Acquiring Urban Environmental Data

Temperature and wetness are the main habitat factors differentiating ecological groups of bryophytes [2,27]. Therefore, the research procedure adopted in this paper was based on the assumption that the key factors differentiating and influencing habitat properties in an urbanized area, and thus the distribution and diversity of epiphytic bryophytes within it, are urban climatic features. These, in turn, differ mainly depending on the characteristics of the urban fabric, which refers to the physical urban environment (e.g., elements, materials, form, scales, etc.) and its socioeconomic and ecological structures. Out of the 30 land-use/land-cover categories in the Urban Atlas database, 19 classes were present in the area of Wrocław, many of which belonged to similar categories in terms of their function or terrain conversion type. Moreover, some of them occupied only small, local areas outside of the study plots. Therefore, leaving them in the analysis would result in an excessive number of meaningless variables. Thus, the classes port areas, airports, fast transit roads and associated land, railways and associated land, and other roads and associated land were combined into one class: transport and communication areas. Moreover, the class industrial and commercial areas was created based on similar principles. The two land-cover categories with the lowest sealing levels of the urban fabric (<10% and 10–30%) were also combined into one. Hence, the potential determinants of the bryophytes analyzed were derived from terrain characteristics, including land-cover and land-usage features, the structure of buildings and vegetation, and terrain relief [23]. Based on our assumption, we used various sources (Table 1) to develop a set of environmental variables for the research area (Table 2), which were direct indicators of climate as well as parameters known from previous theoretical and practical studies to serve as important determinants of urban climate.

**Figure 2:**
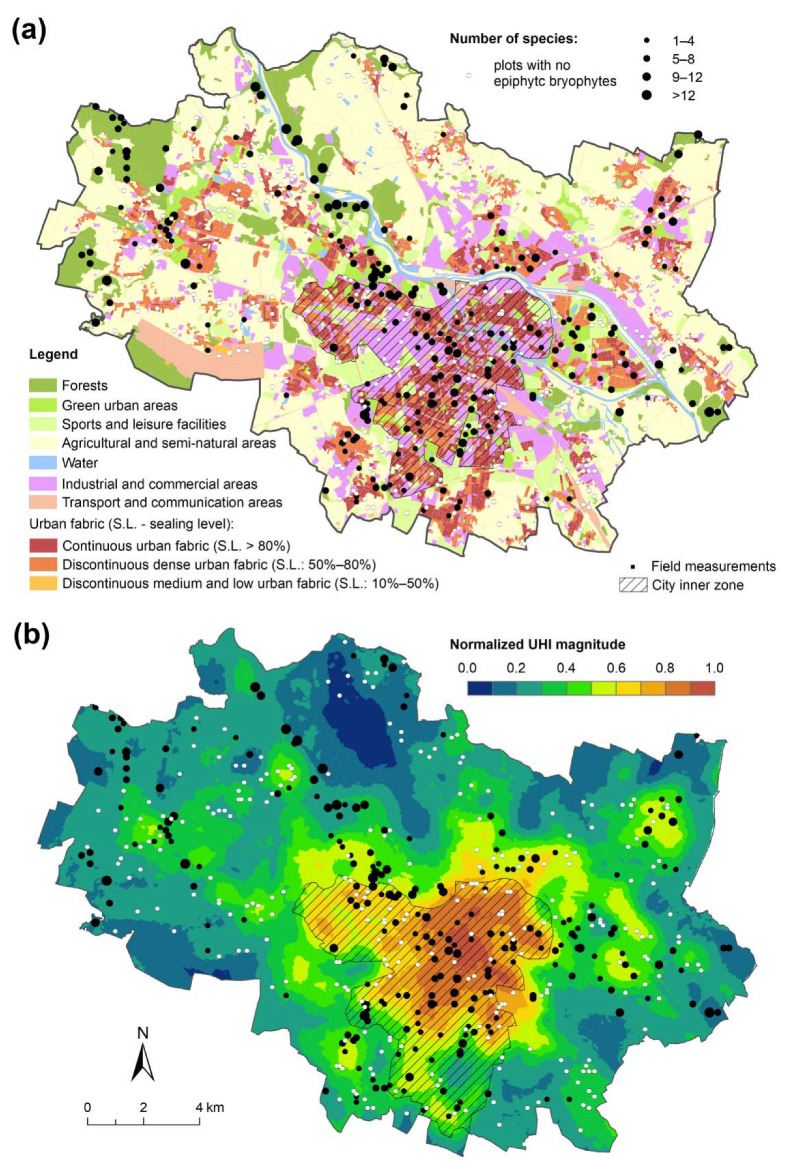
Number of species recorded in the study plots on the background of the urban landscape elements (map (**a**)) and urban heat island magnitude (map (**b**)).

The most common and at the same time the most recognized and best described feature of the climate of urbanized areas is the urban heat island (UHI) manifested as higher air temperature in cities compared to nonurban areas. However, this increase, strongly dependent on urban features and weather conditions, generally occurs systematically from the periphery towards the city center [23,28]. The maps of seven cases of UHI in Wrocław used in this study were prepared by [25]. The UHI episodes were characterized by different magnitudes of the phenomenon; therefore, before averaging, each map was normalized to the range (0, 1). Because the average annual UHI intensity in the city center is about 1 °C [29], the mean normalized UHI map obtained can also be considered an approximation of the average annual UHI in Wrocław (Figure 2b).

The remaining variables selected for analysis were derived from three basic data sources: the Land Use–Land Cover (LULC) database; satellite imagery; and LiDAR-based elevation models (Table 1). The LULC features were analyzed using the high-resolution Urban Atlas 2012. The database, originally containing nearly 30 LULC categories, was generalized by aggregation of similar classes (Figure 1, Table 1). Study plots were then overlaid on the LULC map, and the area was calculated for each class in every plot. As water bodies were present only in a small number of plots (<10%), but the role of water is expected to be significant for the type of vegetation and microclimatic effects, we replaced the water surface index by distance from the water bodies (WATERdi) in the analysis. For this, the Euclidean distance between the water body and the plot centroid was calculated (Table 1). Transport and communication areas were excluded from the analysis as this class covers very different areas (airport, river ports, railroad tracks, different road types, etc.) in terms of physical characteristics, which could give an ambiguous signal quantifying their impact. The analysis included several indicators expressing the diversity of the area in terms of the presence of anthropogenic and natural/seminatural elements. Since their roles in shaping the features of urban climate are known [22,23], they indirectly indicate a dependence on meteorological elements. The greater the anthropogenic land transformation (e.g., sealing level), the higher the air temperature and lower the relative humidity are expected to be.

Another group of urban characteristics was derived from LiDAR scanning data, including a digital elevation model (DEM) and a digital surface model (DSM). The first was a model of bare ground and the other included all objects on it. DEM was used to derive a set of topographic attributes, such as slope inclination (Slp), topographic position index (TPI) [29], and topographic wetness index (TWI) [30] known as determinants of topoclimate [23]. TPI expresses the convexity/concavity of terrain, indirectly indicating areas with a tendency to form or remove pools of cool air. TWI indicates topographically wetter areas, which determine the features of the local climate (cooler, wetter), and potentially affects the type of habitat.

DSM, including data on object height, was used to calculate several straightforward parameters such as mean heights (BUDh and TREEh) and areas covered (BUDAREA and TrCA) by buildings and trees as well as for calculating aerodynamic parameters of the urban environment, i.e., roughness length (z0) [31] and porosity [25]. Roughness length indicates the degree of terrain roughness, with greater values reflecting the resistance of the terrain to moving air mass, which leads to a decrease in wind speed. Simultaneously, porosity may indicate that with severe roughness, moving air mass may flow through obstacles to some extent. Notably, this is mainly a feature of vegetation. The aerodynamic parameters reflect the physical properties of the terrain responsible for modifying air mass movement and turbulent heat exchange [23]. As such, they are an important determinant of the local climate, including the urban heat island [25].

We decided to use satellite data, i.e., Landsat 8 OLI and TIRS data, to characterize the study area in the context of thermal surface features and the distribution of artificial and natural components of the urban fabric. We decided that the satellite imagery used in the analysis should be taken during the field research period (2013–2016) around the middle of the growing season (when trees have fully developed leaves), which is optimally between June and August. During this period, the study area was completely cloudless. Only one image fully met these expectations. It was taken over Wrocław on 11 August 2015 at 9:44 UTC. For this study, a radiometrically and geometrically corrected product (L1T) was used. Atmospheric correction was applied using a single-channel algorithm [32]. Four indices were derived from the satellite data: land surface temperature (LST) [33]; normalized difference vegetation index (NDVI) [34]; normalized difference moisture index (NDMI) [35]; and normalized difference built-up index (NDBI) [36] (Table 2). LST directly shows the level to which the elements of the urban fabric heat up. The vegetation indices are designed to highlight a particular property of vegetation (here chlorophyll [NDVI] and water [NDWI] content), and NDBI highlights built-up areas.

Except for those indices calculated from the LULC maps, the rest of the indices described above were spatially continuous and varied within study plots. Except for the indices calculated from LULC maps, the remainder of the aforementioned indices were spatially continuous and varied within study plots. Apart from the indicators calculated from the LULC maps, the remaining indices were spatially continuous and characterized by internal variability. Therefore, the average level (zonal mean) of each index was assigned to a given study plot. The analyses were performed using ArcGIS 10.2 [37] and SAGA GIS 6.2 software [38].

### 2.5. Variables Used in Analyses

The set of variables used in the analyses is gathered in Table 2. The supply of phorophytes was described for each plot by the total number of tree individuals with a diameter above 10 cm, the number of tree species (NspTr), Shannon–Wiener index for tree species diversity (H’), as well as proportions of individual species in the entire tree stand. Proportions of tree species were counted for species that occurred on at least 10% of plots or those reaching at least 100 individuals totally on all plots. For variables obtained from high-resolution sources (satellite imagery and elevation data), zonal means for every study plot were calculated.

### 2.6. Data Analysis

The Shannon–Wiener index for tree species diversity (H’) was calculated for each plot according to the formula: H′= −Σ (pi × ln pi), where pi = ni/N; ni = the abundance of the tree species is expressed as the number of its individuals and N = the sum of abundances of all tree species is expressed as the total number of individuals. The MVSP v. 3.131 package [39] was used for calculations. Basic statistics were performed using the Statistica v. 13 package [40].

Multivariate analyses were performed using the Canoco 5 package [41]. Data containing the list of epiphytic bryophyte species and the number of records for each study plot were the subject of ordination analyses. Default settings were used in detrended canonical analysis (DCA). Gradient length of 3.95 SD in DCA revealed the unimodal structure of data [42] and, therefore, canonical correspondence analysis (CCA) was used for further study. Sporadically occurring species were down-weighted. CCA analysis was followed by forward stepwise selection of environmental variables and an unrestricted Monte Carlo test with 499 permutations.

## 3. Results

In total, the study recorded 51 bryophyte species (4 liverworts and 47 mosses), including 18 obligate epiphytes, 12 facultative epiphytic–epilithic species, and 13 facultative multisubstrata species (see Appendix A). The remaining bryophyte species appeared to be incidentally epiphytic (i.e., occurred mostly on manmade rocklike habitats and disturbed soil or decayed logs and stumps). Only seven species were recorded in more than 50 study plots: three obligatory epiphytes (*Orthotrichum pumilum* (recorded in 132 plots), *Dicranoweisia cirrata* (67), and *Platygyrium repens* (61)), one facultative epiphytic–epilithic species (*Orthotrichum diaphanum* (176)), and three facultative multisubstrate epiphytes (*Hypnum cupressiforme* (187), *Amblystegium serpens* (141), and *Ceratodon purpureus* (78)) (see Appendix A).

Occurrence of epiphytic bryophytes was recorded in nearly half (237) of the established study plots. The number of different species per plot amounted from 1 to 16. We did not obtain clear and unequivocal distribution patterns for epiphyteless plots or those with various numbers of species based on either the background of differentiated landscape structure elements or the zones of varied normalized UHI magnitude (Figure 2a,b). Both epiphyteless and epiphyte-poor plots were situated in the dense built-up zone as well as other zones such as urban forests (Figure 2b). However, plots located within near-natural forests generally had higher numbers of epiphytic bryophyte flora.

A total of 69 tree species were registered in all study plots with bryophyte occurrences; the most frequent were *Quercus robur* (1591 individuals recorded in 91 plots), *Carpinus betulus* (1148/66), *Acer platanoides* (1065/128), *Tilia cordata* (819/104), *Robinia pseudoacacia* (785/111), *Fraxinus excelsior* (725/95), *Picea abies* (540/82), *Betula pendula* (485/78), and *Acer pseudoplatanus* (482/86). However, bryophytes were found on 41 tree species (59.4%) in total and only 7 of these tree species were frequently colonized by bryophytes: *Fraxinus excelsior* (139 trunks—19.0% of the all specimens of that species recorded in the plots), *Betula pendula* (72—14.8%), *Populus* x *canadensis* (90—12.3%), *Quercus robur* (174—10.9%), *Robinia pseudoacacia* (80—10.2%), *Tilia cordata* (76—9.2%), and *Acer platanoides* (93—8.7%). Another 20 tree species were occupied sporadically, with the number of tree individuals colonized less than 10 for each.

**Figure 3:**
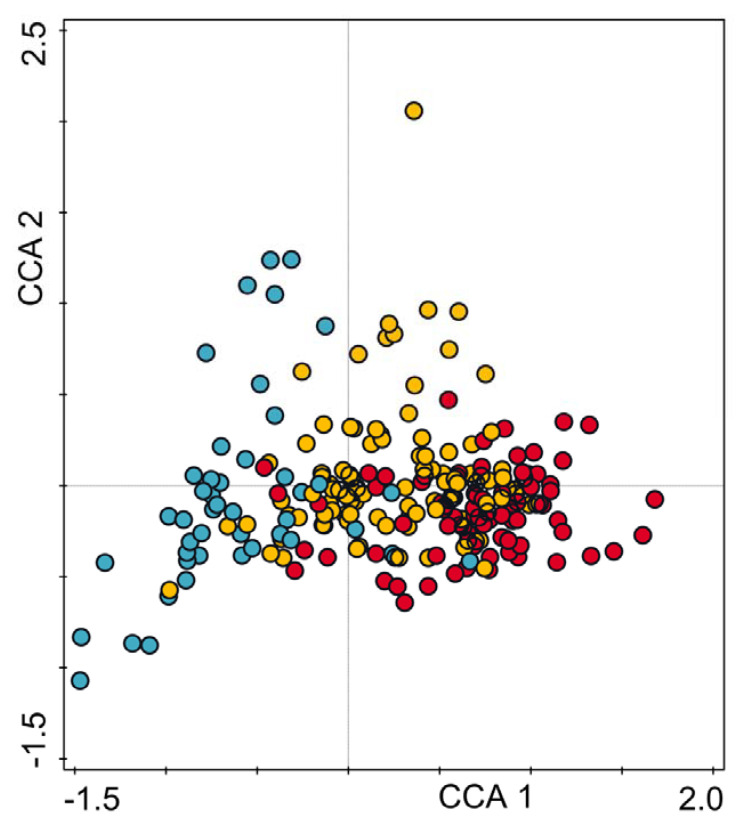
Ordination of study plots. Plots belonging to the dense built-up zone are marked in red, intermediate zone with scattered built-up fabric in orange and suburban forests in blue. Classification of plots is based on the analysis of the structural landscape elements. Compare Figure 1.

The first two CCA axes explained 22.0% of the variance of species data (axis 1: 15.6%; axis 2: 6.4%) and 64.7% of the variance of fitted response data (axis 1: 45.9%; axis 2: 18.8%). All canonical axes turned out to be significant (F = 5.6406; *p* = 0.0020). Study plots are well-separated along the first CCA axis in terms of their distribution in the urban space (Figure 3). Those from the inner city with continuous built-up areas (hatched area on maps, see Figure 1 and Figure 2a) gathered at the maximum of the first axis. Plots from suburban forests and parks had the minimum values and those located in the intermediate localities mostly occupied the mid sector of the first CCA axis.

**Figure 4:**
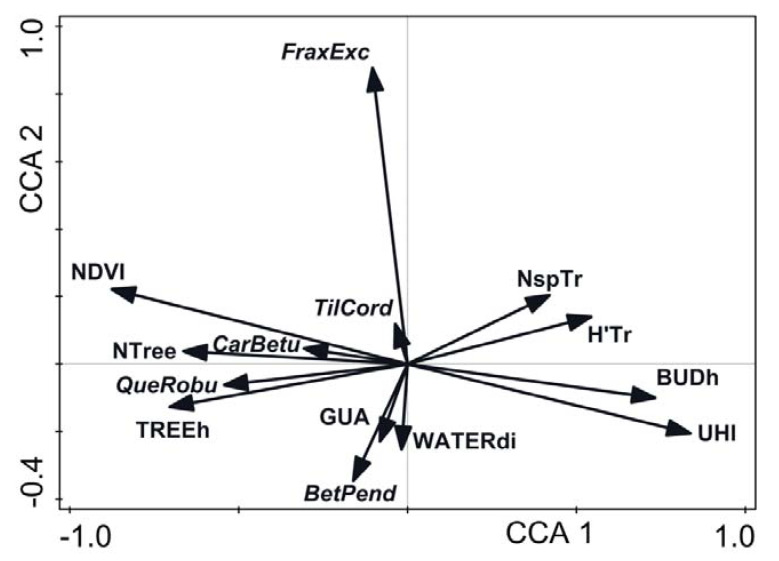
CCA environmental variables ordination. For abbreviations of variables see Table 2. Labels in italics denote tree species names: *Fraxinus excelsior*, *Tilia cordata*, *Carpinus betulus*, *Quercus robur*, and *Betula pendula*.

Environmental factors which significantly influenced the epiphytic bryophytes’ distribution after forward selection of explanatory variables are shown in Figure 4. The normalized difference vegetation index (NDVI) and the urban heat island (UHI) were the strongest variables correlated with the first axis (r = −0.7909, 0.7566, respectively). The share of *Fraxinus excelsior* in the tree stands appeared to be the strongest variable positively correlated with the second CCA axis (FraxExc, r = 0.6655). However, an analysis of the conditional effects (Table 3) revealed that, acting simultaneously (conditional term effects), the strongest effects on the epiphytic bryophytes distribution were the following: NDVI, share of *Fraxinus excelsior* trees (FraxExc), tree mean height (TREEh), and nearest water body distance (WATERdist). The urban heat island (UHI) appeared to be the last significant variable and mean building height (BUDh) turned out to be not significant.

**Figure 5:**
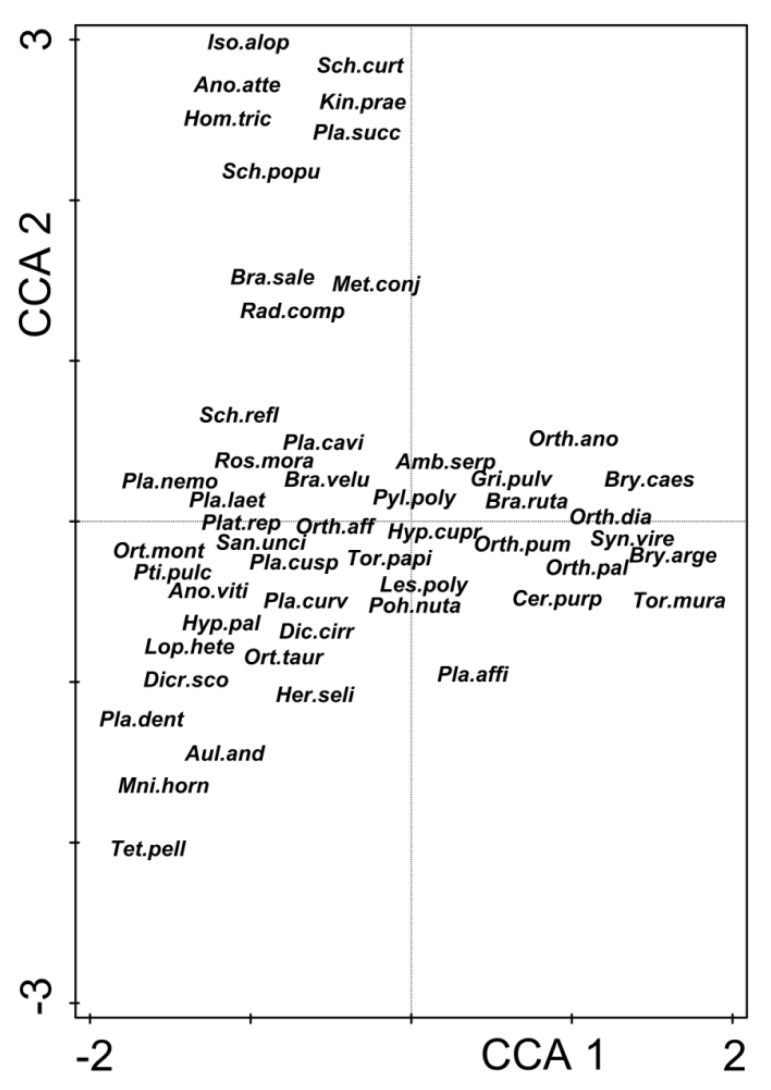
CCA ordination diagram for epiphytic bryophyte species. For a list of species name abbreviations, see Appendix A.

**Figure 6:**
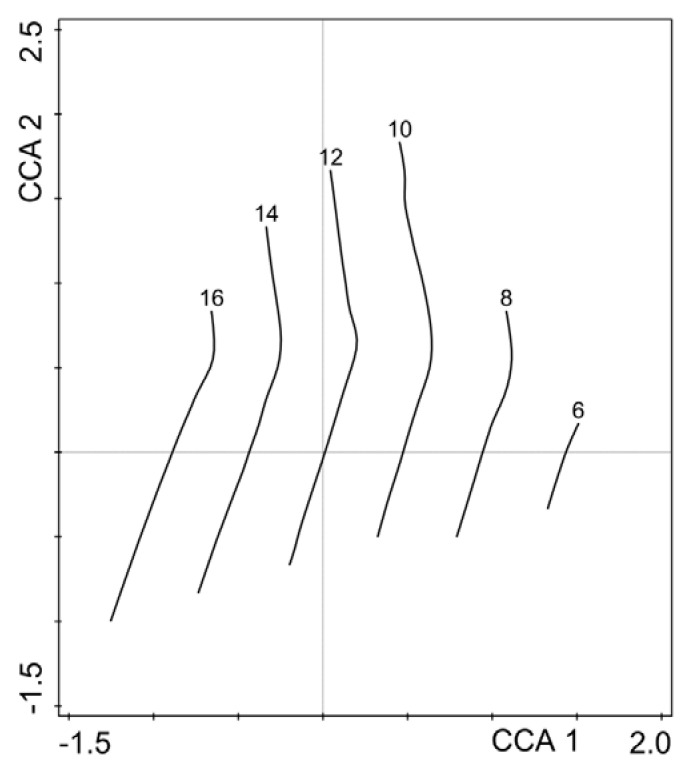
Isolines indicating epiphytic bryophyte species richness through the CCA ordination space. Loess smoothing model was used.

Species positions in the CCA space (Figure 5) are scattered along both axes because of specific reactions to environmental factors and host tree species shares, as shown in the diagram (compare Figure 4). Within the sector of the maximum values of the first axis were gathered species relatively tolerant to high temperatures, dryness, and preferring more sunny sites. Here, belong the considerably thermophytic and xerophytic *Orthotrichum diaphanum* (Orth.dia) and meso- to considerably thermophytic and xerophytic *Syntrichia virescens* (Syn.vire). On the lefthand side of the figure are species typical of forest habitats with preferences for lower temperatures and higher humidity and shade, such as *Platygyrium repens* (Plat.rep), *Hypnum pallescens* (Hyp.pal), *Plagiothecium laetum* (Pla.laet), *Ptilidium pulcherrimum* (Pti.pulc), and *Sciuro*-*hypnum reflexum* (Sch.refl). Some species, recorded mostly in riparian ash forests, clustered within the highest values range of the second axis, corresponding with the vector of *Fraxinus excelsior* share. Typical epiphytes belonged here, including *Isothecium alopecuroides* (Iso.alop), *Anomodon attenuatus* (Ano.atte), *Homalia trichomanoides* (Hom.tri) and *Sciuro-hypnum populeum* (Sch.popu). These species prefer subneutral substrates and were found mainly on the trunks of ashes. On the other hand, this group also encompasses species usually inhabiting the forest bottom but not tree trunks, such as *Kindbergia praelonga* (Kin.prae) and *Sciuro-hypnum curtum* (Sch.curt). Isolines in Figure 6 apparently indicate that the epiphytic bryophyte species richness of the study plots is negatively correlated with the first axis of the CCA. It means that the species richness of epiphytic bryophytes grew in an inverse relationship to the urbanization gradient from the city center to the suburban forests (compare Figure 4 and Figure 6). Significant Spearman correlations were not found between the number of epiphytic bryophytes in plots and the number of tree species in the plot (r = −0.0459, *p* = 0.4820), nor for the Shannon-Wiener diversity index calculated for tree species (r = −0.1198, *p* = 0.0655).

## 4. Discussion

At the start of our study, we assumed that the urban heat island (UHI) resulting from the structural diversity of the urban landscape is the main factor influencing the distribution and richness of epiphytic bryophyte species within the urban space. That hypothesis was generally positively verified as the first axis of the CCA reflects the urbanization gradient shaping the UHI. Seven epiphytic bryophyte species showed a positive response to the urban heat island, while more than 20 showed a negative response. The latter were mesophytic species widespread in forests and large urban parks in Central Europe [12,13,43]. Three of these were the obligatory epiphytes, *Anomodon attenuatus*, *A. viticulosus*, and *Homalia trichomanoides*, which are considered to be primeval forest relics in the Western Carpathians [44]. Our studies showed that the species richness of epiphytic bryophytes decreased visibly along the increasing gradient of urbanization, from suburban forests to the city center. Of the seven species that showed a positive reaction to the UHI, two (*Orthotrichum diaphanum* and *Syntrichia virescens*) have hitherto been characterized as species tolerant to high temperatures and dryness [27].

However, the effect of the UHI interacts with the influences of other environmental factors: such as, NDVI, the proportion of *Fraxinus excelsior* in tree stands, mean tree height, and distance to the nearest water body. In our study, NDVI, which was significantly negatively correlated with the UHI, appeared to be the variable with the strongest effect regardless of whether it was analyzed separately or against the background of other factors. The effect of the UHI was also modified by the distance to the nearest water body (WATERdi) and the occurrence of certain tree species, particularly ash (*Fraxinus excelsior*). These observations explain the positive role of riparian forests with a high number of ash trees in maintaining a large number of epiphytic bryophytes. These plants, which are sensitive to drought stress, find higher air humidity and appropriate host trees in such communities. Our findings are in line with the results of [3,4], who revealed that changes in microclimate caused by UHIs affected bryophyte species richness in historical Japanese moss gardens. In a study carried out in Düsseldorf, Germany, Stapper and Kricke [45] found a strong negative effect of relative nocturnal temperature and eutrophication—caused mainly by transport—on the diversity and frequency of epiphytic lichens and bryophytes. It should also be emphasized that the bryophytes in their study apparently preferred sites with lower relative nocturnal temperatures as compared to lichens.

However, we are aware of the limitations of our research. One of these limitations is that we were unable to estimate the effect of pollution background at the scale of the entire city. Therefore, we consciously omitted spatial variability in terms of the degree of urban environmental pollution; however, this factor was taken into account in some studies devoted to the distribution of bryophytes within urbanized areas (e.g., [45,46,47]). Larsen et al. [46], who investigated the distribution of epiphytic lichens and bryophytes on oaks in London, UK, found a negative effect of transport-related pollution and bark acidity. On the other hand, those authors did not exclude the possibility that humidity and temperature could also play an important role.

Our study showed that one of the strongest factors positively affecting both epiphytic bryophyte species richness and diversity was mean tree height. This parameter, obtained through remote sampling, can be used as a measure of the tree size analogous to the diameter at breast height (DBH) measurement collected in the field. Many authors have highlighted that the frequency of large trees is one of the most important factors affecting the number, diversity, and sometimes the composition and abundance of epiphytic bryophyte species in forests [13,15,16,17,43,48,49]. This explanation regarding the importance of large trees for epiphytic bryophyte species richness seems to be rather obvious. According to Fritz et al. [50], the crucial factors include a larger trunk area and longer time available (as suitable for colonization by bryophytes).

In the present study, we did not find any significant correlation between tree species richness and diversity (expressed as Shannon–Wiener index H’) and the richness of epiphytic bryophyte species. This contradicts some studies that showed the positive effect of tree species diversity on the number of bryophyte species in forests [14,15,16,17]. However, a proportion of *Fraxinus excelsior* in tree stands was found to be among the variables with the strongest effects on epiphyte distribution and species richness in Wrocław. The importance of ash trees as a preferred phorophyte for many epiphytic bryophytes in southern Britain was emphasized by [51,52]. Thus, the presence of certain tree species is advantageous for bryophytes since some of the host tree species seem to influence epiphyte richness and diversity in Wrocław. *Quercus* spp., *Carpinus betulus*, and *Fraxinus excelsior* were listed among the tree species carrying the highest number of epiphytic bryophytes in forests by various authors [14,15,16]. This is also in line with our results, but only in terms of forests and suburban areas. Within the inner densely built-up zone, trunks of these tree species were colonized very rarely despite their rather numerous presences, and the number of epiphytic species found was small. This sparse occurrence of bryophytes on the trunks of tree species characteristic of natural forests may be a result of the higher diversity of tree species in the built-up areas, where higher numbers of introduced tree species occur. Some alien tree species seem to be easier for settling on by bryophytes compared to native trees [53,54].

Within the studied area, we found the highest species richness and diversity of epiphytic bryophytes in suburban forests, especially those with deciduous or mixed stands, which indicates at least some near-natural features, particularly a differentiated space structure of community and age structure of tree populations. These results may be seen as a general indication of management conducive to maintaining a rich epiphytic bryophyte flora. They are also in line with the suggestions of various authors concerning management measures, such as increasing the share of deciduous species, maintaining old, large trees, creating a spatial structure with differentiated microsites offering heterogeneous light conditions, and maintaining tree species diversity [14,16,17,43]. Maintaining green areas within the less dense urban fabric with differentiated vegetation, but also with as many old deciduous trees as possible, may be beneficial to preserving both the diversity and richness of epiphytic bryophyte flora and comfortable climatic and aesthetic values for urban inhabitants as important factors of the healthy environment. This is one more reason to maintain the heterogeneity of urban green spaces by diverse management measures, which was also postulated by [20]. Cities as peculiar modern biodiversity refugia need further scientific study and development of conservation methods. Studies devoted to urban bryophytes facing the increasing effect of ever hotter heat islands may be an important part of such programs.

## 5. Conclusions

Among the 51 epiphytic bryophyte species found in the urban area of Wrocław, mesic obligatory epiphytes and facultative epiphytes dominated. Urban climate factors related to the structural landscape elements and features of tree stands were decisive for the distribution and species richness of the epiphytic bryophytes. The normalized difference vegetation index (NDVI) had the strongest positive effect on the distribution and richness of the epiphyte bryophytes; tree height had a lesser effect. The intensity of the heat island (UHI) had a negative impact on both parameters. Additionally, the share of the European ash *Fraxinus excelsior* in the overall tree count, distance from a water body (WATERdi), and tree species diversity index (H’Tr) were the strongest factors affecting the distribution of epiphyte bryophytes. However, we did not find any significant relationship between tree species diversity, expressed as the Shannon–Wiener index (H’) and the richness and diversity of epiphytic bryophyte species.

Maintaining green areas within the less dense urban fabric with differentiated vegetation, but with as many old deciduous trees as possible, may be beneficial to both preserving diversity and richness of the epiphyte bryophyte flora as well as keeping comfortable climatic and aesthetic values for urban inhabitants.

Our research was carried out in a big European city located in the temperate climate zone, with differentiated structures of urban landscape. We treat it as a case study, but one can assume that our results and conclusions may concern other urban areas of similar properties.

## Figures and Tables

**Table 1:** Geographic data used in the study.

Source Dataset	Data Model	Minimum Mapping Unit	Spatial Resolution (Pixel Size)	Year of Origin	Coordinate Reference Datum/ Projection EPSG Code
Land use/land cover: Urban Atlas 2012 (https://land.copernicus.eu/local/urban-atlas/, accessed on day 22 September 2017)	Vector	2500 m^2^	-	2012	ETRS89/LAEA EPSG: 3035
Satellite imagery: Landsat 8 L1TP product, 11 August 2015: LC08_L1TP_190024_20150811_20170406_01_T1 (https://earthexplorer.usgs.gov/, accessed on 22 September 2017)	Raster	-	30 × 30 m	2015	WGS84/UTM33N EPSG: 32633
LiDAR data: Digital Elevation Model, Digital Surface Model ISOK project, Poland (http://www.gugik.gov.pl/projekty/isok/produkty, accessed on 15 April 2013)	Raster	-	1 × 1 m	2011	ETRS89/Poland CS92 EPSG: 2180
Urban Heat Island data: [28]	Raster	-	25 × 25 m	Measured: 2001-2002 Modelled: 2012	ETRS89/Poland CS92 EPSG: 2180

**Table 2:** Explanatory variables used in the epiphyte bryophyte species richness and diversity analyses. See Section 2.4 for broader explanation.

Variable	Abbreviation	Unit	Mean	SD	Range
In-field sampled data					
Number of trees	NTree	number	72.01	10.67	5–524
Shannon-Wiener diversity index for trees	H’Tr	–	1.73	0.27	0.14–2.93
Shannon-Wiener evenness index for trees	J’Tr	–	0.80	0.02	0.16–1.0
Number of tree species	NspTr	number	9.50	1.33	2–27
Land use/land cover data					
Agricultural and seminatural areas	AgrSn	m^2^	1157.1	0	0–10,000
Continuous urban fabric >80%	CUF	m^2^	1902.8	0	0–10,000
Discontinuous dense urban fabric 50–80%	DDUF	m^2^	1355.7	0	0–9985
Discontinuous medium density urban fabric 30–50%	DMDUF	m^2^	61.4	0	0–5388
Forests	FOR	m^2^	1609.2	0	0–10,000
Green urban areas	GUA	m^2^	1182.2	0	0–10,000
Industrial commercial public military and private units	IndMil	m^2^	1364.4	0	0–10,000
Sports and leisure facilities	SLF	m^2^	418.0	0	0–10,000
Nearest Water Body Distance	WATERdi	m	950.3	118.6	1–3575
Satellite imagery derivatives					
Land Surface Temperature	LST	°C	37.9	0.48	32.2–45.5
Normalized Difference Vegetation Index	NDVI	–	0.25	0.04	−0.005–0.402
Normalized Difference Moisture Index	NDMI	–	0.13	0.05	−0.023–0.2862
Normalized Difference Built-up Index	NDBI	–	−0.13	0.05	−0.2862–0.0230
LiDAR scan derivatives					
Built-up Area	BUDAREA	m^2^	1034.0	0	0–8100
Buildings height	BUDh	m	7.36	1.58	0–33.4
Tree Covered Area	TrCA	m^2^	3553	1117.8	1–9846
Trees Mean Height	TREEh	m	9.35	1.83	2.51–22.11
Roughness length	z0	m	0.97	0.27	0–4.893
Porosity	POROSITY	–	0.79	0.10	0–0.99
Altitude	Alt	m a.s.l	119.2	0.91	110–135
Slope	Slp	deg.	1.97	0.80	0.381–12.440
Topographic Position Index	TPI	–	0.02	0.03	−0.899–2.616
Topographic Wetness Index	TWI	–	8.45	0.37	5.408–12.421
Climatological data					
Urban Heat Island	UHI		0.470	0.01	0.058–0.948

**Table 3:** Simple term and conditional term effects of the studied variables. For abbreviations of urban environmental variables see Table 1. Tree species name abbreviations: Ace.plat—*Acer platanoides*, Ace.pseu—*Acer Acer pseudoplatanus*, Bet.pend—*Betula pendula*, Car.betu—*Carpinus betulus*, Frax.exc—*Fraxinus excelsior*, Pic.abie—*Picea abies*, Que.robu—*Quercus robur*, Til.cord—*Tilia cordata*.

Variable	Explains %	Pseudo-F	*p*	P (adj)
Simple Term Effects				
NDVI	12.4	33.2	0.002	0.03992
UHI	11.4	30.2	0.002	0.03992
BUDh	9.3	24.2	0.002	0.03992
BUDarea	8.7	22.4	0.002	0.03992
TREEh	8.4	21.4	0.002	0.03992
NTree	7.4	18.7	0.002	0.03992
POROSITY	6.6	16.6	0.002	0.03992
Frax.exe	5.5	13.6	0.002	0.03992
Que.robu	5.4	13.3	0.002	0.03992
H’Tr	5.1	12.8	0.002	0.03992
Ace.pseu	3.7	9.1	0.002	0.03992
NspTr	3.4	8.3	0.002	0.03992
Ace.plat	3.1	7.6	0.002	0.03992
Rob.pseu	2.4	5.8	0.002	0.03992
Car.betu	2.4	5.8	0.002	0.03992
WATERdi	2.0	4.8	0.002	0.03992
GUA	1.9	4.4	0.002	0.03992
Bet.pend	1.5	3.7	0.016	0.31936
Til.cord	0.8	1.9	0.047	0.95808
Pic.abie	0.6	1.4	0.120	1.00000
Conditional Term Effects				
NDVI	12.39	33.2	0.002	0.03992
Frax.exe	5.28	15.0	0.002	0.03992
TREEh	2.86	8.4	0.002	0.03992
WATERdi	2.38	7.2	0.002	0.03992
GUA	1.45	4.4	0.002	0.03992
Bet.pend	1.14	3.5	0.002	0.03992
Car.betu	1.03	3.2	0.004	0.03992
NTree	1.05	3.3	0.002	0.03992
H’Tr	0.99	3.1	0.002	0.03992
Que.robu	0.72	2.3	0.002	0.03992
UHI	0.73	2.4	0.002	0.03992
Til.cord	0.67	2.2	0.008	0.15968
BUDh	0.51	1.6	0.040	0.79840
NspTr	0.48	1.6	0.046	0.91816
POROSITY	0.44	1.4	0.096	1.00000
BUDarea	0.43	1.4	0.100	1.00000
Ace.pseu	0.41	1.3	0.176	1.00000
Ace.plat	0.37	1.2	0.200	1.00000
Rob.pseu	0.33	1.1	0.330	1.00000
Pic.abie	0.28	0.9	0.547	1.00000

## Data Availability

Not applicable.

## Appendix A

**Table A1:** List of the bryophyte species recorded with some information of their occurrence and their name abbreviations used in the CCA analysis. Explanations of symbols: **—obligatory epiphyte, *e—facultative, epiphytic–epilithic, species, *m—facultative, multisubstrata epiphyte, ○—incidentally epiphytic species.

Species Name	Abbreviation	Number of Plots in Which the Species Was Recorded
*Amblystegium serpens* *e	Amb.serp	141
*Anomodon attenuates* **	Ano.atte	6
*Anomodon viticulosus* **	Ano.viti	1
*Aulacomnium androgynum* ○	Aul.and	16
*Brachytheciastrum velutinum* *m	Bra.velu	30
*Brachythecium rutabulum* *m	Bra.ruta	86
*Brachythecium salebrosum* *m	Bra.sale	11
*Bryum argenteum* ○	Bry.arge	33
*Bryum caespiticium* ○	Bry.caes	2
*Ceratodon purpureus* *m	Cer.purp	78
*Dicranoweisia cirrata* **	Dic.cirr	67
*Dicranum scoparium* *e	Dicr.sco	24
*Grimmia pulvinate* ○	Gri.pulv	3
*Herzogiella seligeri* ○	Her.seli	2
*Homalia trichomanoides* **	Hom.tric	8
*Hypnum cupressiforme* *e	Hyp.cupr	187
*Hypnum pallescens* **	Hyp.pal	18
*Isothecium alopecuroides* **	Iso.alop	1
*Kindbergia praelonga* *m	Kin.prae	2
*Leskea polycarpa* **	Les.poly	13
*Lophocolea heterophylla* *e	Lop.hete	23
*Metzgeria furcate* **	Met.conj	2
*Mnium hornum* *m	Mni.horn	2
*Orthodicranum montanum* **	Ort.mont	15
*Orthodicranum tauricum* **	Ort.taur	12
*Orthotrichum affine* **	Orth.aff	49
*Orthotrichum anomalum* ○	Orth.ano	5
*Orthotrichum diaphanum* *e	Orth.dia	176
*Orthotrichum pallens* *e	Orth.pal	22
*Orthotrichum pumilum* **	Orth.pum	132
*Plagiomnium affine* *m	Pla.affi	2
*Plagiomnium cuspidatum* *m	Pla.cusp	3
*Plagiothecium curvifolium* *m	Pla.curv	2
*Plagiothecium denticulatum* *m	Pla.dent	1
*Plagiothecium laetum* **	Pla.laet	7
*Plagiothecium nemorale* *m	Pla.nemo	2
*Platygyrium repens* **	Plat.rep	61
*Pohlia nutans* *m	Poh.nuta	2
*Ptilidium pulcherrimum* **	Pti.pulc	1
*Pylasia polyantha* *e	Pyl.poly	3
*Radula complanate* **	Rad.comp	11
*Rosulabryum moravicum* *e	Ros.mora	32
*Sanionia uncinate* *e	San.unci	2
*Sciuro-hypnum reflexum* *e	Sch.refl	1
*Sciuro-hypnum curtum* *m	Sch.curt	1
*Sciuro-hypnum populeum* *e	Sch.popu	4
*Syntrichia virescens* *e	Syn.vire	37
*Tetraphis pellucida* ○	Tet.pell	2
*Tortula latifolia* **	Tor.lati	1
*Tortula muralis* ○	Tor.mura	2
*Tortula papillosa* **	Tor.papi	1
